# Resource Selection by Wild and Ranched White-Tailed Deer (*Odocoileus virginianus*) during the Epizootic Hemorrhagic Disease Virus (EHDV) Transmission Season in Florida

**DOI:** 10.3390/ani11010211

**Published:** 2021-01-16

**Authors:** Emily T.N. Dinh, Jeremy P. Orange, Rebecca M. Peters, Samantha M. Wisely, Jason K. Blackburn

**Affiliations:** 1 Spatial Epidemiology & Ecology Research Laboratory, Department of Geography, University of Florida, Gainvesille, FL 32611, USA; emilydinh@ufl.edu (E.T.N.D.); jporange2@ufl.edu (J.P.O.); 2 Emerging Pathogens Institute, University of Florida, Gainesville, FL 32611, USA; 3 Florida Fish and Wildlife Conservation Commission, Gainesville, FL 32601, USA; Becky.Peters@myfwc.com; 4 Department of Wildlife Ecology and Conservation, University of Florida, Gainesville, FL 32611, USA; wisely@ufl.edu

**Keywords:** epizootic hemorrhagic disease virus, white-tailed deer, resource selection function, Florida

## Abstract

**Simple Summary:**

Epizootic hemorrhagic disease virus is transmitted by *Culicoides* midges and causes serious disease in wild and privately ranched white-tailed deer (*Odocoileus virginianus*) in the United States. The U.S. deer ranching industry is fast growing and generates an estimated ~USD 8 billion annually. In Florida, there are over 400 registered deer farms, and virus rates are high among these populations. While vaccines for the virus are becoming available, many farms have large hunting preserves, where safely capturing deer is difficult. At the same time, these farms are situated in proximity to wild deer populations, and both populations are at risk. We studied habitat selection in ranched deer within a ~180 ha high-fenced preserve. We GPS-collared deer in the hunting preserve and nearby state-managed lands to compare habitat selection. During 2016, we collected GPS data from 15 ranched and eight wild deer and built resource selection function models. These models suggest ranched deer select habitats more likely to support several midge species that transmit the virus compared to wild deer. These differences in habitat use may partially explain previously confirmed higher rates of disease exposure in the ranched deer. Our results may inform ranch land management strategies that reduce midge–deer contact.

**Abstract:**

Epizootic hemorrhagic disease virus (EHDV) causes serious disease in wild and privately ranched white-tailed deer (*Odocoileus* *virginianus*) in the United States. In Florida, there is high EHDV prevalence, yet no treatments. There are few management strategies for the disease due to limited knowledge of virus–vector–host interactions. We conducted a telemetry study on white-tailed deer to examine resource use by wild and ranched animals in the Florida panhandle during the 2016 transmission risk period. We built generalized linear mixed models (GLMMs) to estimate resource selection and map habitat preferences for wild and ranched deer in the study area to reveal how second-order selection may relate to higher disease prevalence in ranched deer. Wild deer preferred areas closer to tertiary roads and supplementary food sources but farther from permanent water. Ranched deer selected bottomland mixed forest and areas closer to tertiary roads, supplementary food sources, and permanent water. Ranched deer behaviors may increase the likelihood of EHDV vector encounters, as these deer selected preferred habitats of several putative vector species, which may increase vector blood meal success and viral transmission risk. Disparate resource selection behaviors may be a factor in observed differential EHDV exposure risk between ranched and wild white-tailed deer in Florida.

## 1. Introduction

Understanding what specific resources animals select (or avoid) can elucidate how wildlife may encounter competent vectors and that would result in transmission [1,2]. Revealing vital links between animal behavior and disease transmission can allow us to predict the spatial distribution of hosts, pathogens, and vectors to better identify areas of high transmission risk [2,3]. If areas of high potential disease transmission risk are identified, then more appropriate disease intervention strategies can be developed. Several studies have quantified and mapped risk for indirectly transmitted bacterial pathogens in cervids by characterizing resource selection behavior. For instance, Proffit et al. [3] showed that female elk, *Cervus canadensis*, select privately owned lands during the brucellosis transmission risk period, increasing commingling with livestock, which then increases pathogen spillover risk. Morris et al. [2] estimated resource selection by male elk and compared areas of high elk selection and high anthrax risk to assess disease potential. Similar research is needed to understand how ungulate resource selection relates to indirectly transmitted viruses.

Epizootic hemorrhagic disease virus (EHDV), vectored by *Culicoides* biting midges (Diptera: Ceratopogonidae), is the causative agent of epizootic hemorrhagic disease (EHD), which affects wild and domesticated ruminants [4]. White-tailed deer (WTD; *Odocoileus virginianus*) in North America often survive EHDV, but the disease can lead to secondary infections that may result in extensive organ damage and disability through lameness and emaciation from loss of appetite [5,6,7,8,9]. The deer farming industry generates an estimated USD 8.0 billion in annual economic activity and is one of the fastest growing industries in the rural United States [10]. Most farms produce or manage WTD [10], so infectious diseases such as EHDV present a major industry challenge. Animals may be confined in pens with relatively small areas, ranging within a high-fenced property, or both. Farmers, nationwide, often also raise a large variety of non-native ungulate species [11,12,13].

EHDV is widespread throughout the U.S., but there are presently no treatments for ranched animals and few control strategies [4,6]. There are currently no licensed vaccines for the virus in the U.S. and available autogenous vaccines are typically designated for use in the deer herd from which the virus isolate was recovered. Preliminary data indicate that available vaccines do not induce a significant, robust antibody response in WTD inhabiting a high-fenced outdoor enclosure [8,14]. Since the vaccines are injectable, this disease control strategy is unsuitable for free-ranging animals on large properties, as has been noted for other vaccine preventable infectious pathogens [15]. There are few other disease management options, as there is limited knowledge of virus–vector–host interactions [4,16].

After examining serological data from 27 different free-ranging ranched and 53 wild WTD in the same study areas as the study presented here, we found that ranched animals had higher seroprevalence and antibody titers against EHDV than wild deer [17]. We detected 23–70% seropositivity for three different serotypes of EHDV in our two-year serological study on wild WTD [17]. In each of the two years, ranched deer had significantly higher seroprevalence of the predominant serotype of EHDV than the adjacent wild deer population. Higher EHDV exposure in ranched deer may be due to many differences including host or vector density, host immunological status, movement behavior, or resource selection in the two groups. As part of an effort to understand epidemiological factors that drive transmission, we took a comparative approach to discovering major sources of dissimilar resource selection between ranched and wild deer during the EHDV transmission season that possibly contribute to the differential exposure to EHDV recently reported [17]. For this study, we define the EHDV transmission period as May–October. As recently summarized, hemorrhagic disease cases (which may also include Bluetongue Virus) are reported June–November, with the peak between August–October [18].

A resource selection function (RSF) is any model that attempts to determine what resources animals prefer by yielding values proportional to the probability of use for a resource unit [19,20]. When resources are used disproportionately to their availability, the animal is considered to be selecting, or avoiding, that resource [20]. This study is focused on second-order selection [21]. Here, we aimed to better comprehend why exposure was higher in ranched versus wild deer in the panhandle of Florida by comparing resource selection amongst ranched and wild deer near each other but separated by high fences. Our research questions were (1) what differences, if any, were there in resource selection between ranched and wild deer during the 2016 EHDV season and (2) which resources did ranched and wild deer select for or avoid. We aimed to build a model of resource selection applicable to both wild and ranched populations, then we mapped and compared resource preferences across the study area for both groups of deer. Next, we sought to connect our findings on deer behavior to implications on EHDV transmission, prevalence, and control.

## 2. Materials and Methods

### 2.1. Study Areas

**Figure 1:**
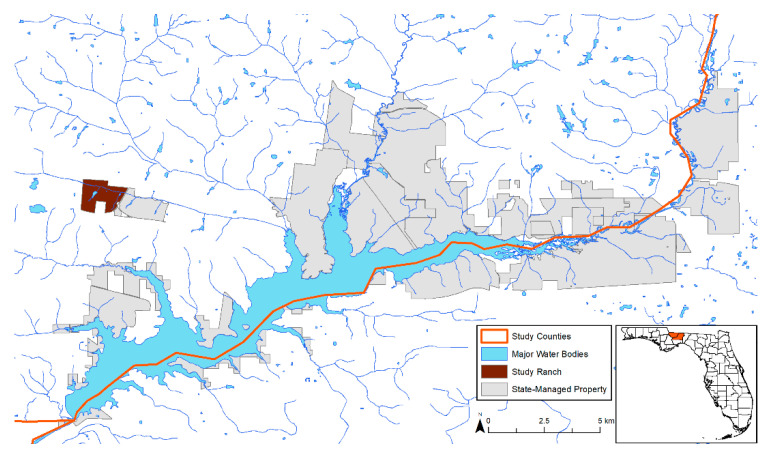
Location of the private study ranch and nearby state-managed properties where wild white-tailed deer (*Odocoileus virginianus*) were studied in Gadsden and Leon counties, Florida.

**Figure 2:**
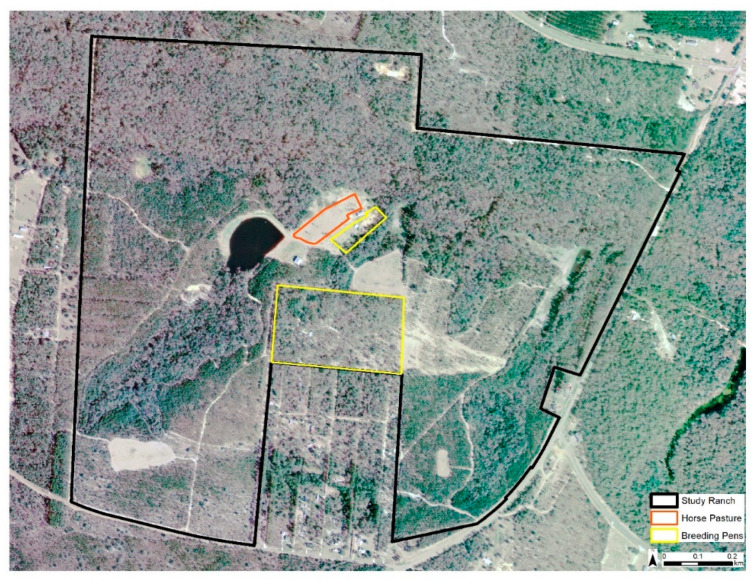
Detailed map of the study private deer ranch in Gadsden County, Florida.

Our study on ranched deer focused on a free-ranging herd within an approximately 200 ha privately owned, high-fenced deer ranch in Gadsden County, Florida (Figure 1). The property was separated into a ~180 ha hunting preserve and a ~20 ha enclosed WTD breeding center. The breeding center included 10 high-fenced WTD breeding pens that occupied ≅8.5 ha total and one pen that occupied ≅0.8 ha that free-ranging animals could not access (Figure 2). At the time of this study, there were 130–150 free-ranging WTD in the hunting preserve portion of the ranch along with ≅157 individual exotic cervids and bovids of 13 different species [22], yielding a population density of approximately 1.48 animals/ha. The breeding pens consisted of improved pastures seeded with Florida native and Bahia grasses. The dominant landscape on the property was hardwood hammock. Upland short leaf pine species such as lobolly (*Pinus taeda*) were also a prominent feature on the property. The land was managed with food plots and multiple supplemental protein feeders filled regularly by ranch staff. The primary management objective of the ranch was cervid propagation. In our previous serological study, we found that 33–100% (depending on the virus serotype) of the free-ranging ranched WTD studied were seropositive for EHDV over a two-year study period [17].

Wild deer in this study inhabited state-managed properties within Gadsden and Leon counties, near the study ranch (Figure 1), at an estimated density of 0.08 animals/ha [17]. These properties are managed by the Florida Fish and Wildlife Conservation Commission (FWC) and the Florida Forest Service (FFS). Their management objectives included providing opportunities for human recreation, timber harvest, and environmental services. The landscape on these properties consisted of hardwood hammock, mesic flatwoods, upland pine (*P. elliottii* and *P. palustris*), and sandhill. The pine on the state-managed properties was either naturally regenerated or in even-aged plots.

### 2.2. Capture, Handling, and Telemetry Data Collection

On the study ranch, we captured 8 male and 7 female WTD (*n* = 15) in April–June 2016 using chemical immobilization delivered via projectile darts loaded with 1.5 to 2.0 cc pre-mixed Butorphanol Tartrate–Azaperone Tartrate–Medetomidine HCl (BAM; Wildlife Pharmaceuticals, Windsor, CO, USA). We fitted ranched deer with GPS collars (model 3300L or 3300S, Lotek, Newmarket, ON, Canada; or model G2110E2 (NeoLink), ATS, Isanti, MN, USA.). BAM was reversed with 0.5 mL Naltrexone HCl and Atipamezole HCL at double the dosage of BAM delivered via intramuscular injection in the shoulder or hindquarter. Similarly, on state-owned properties, we captured 3 male and 5 female WTD (*n* = 8) in May–July 2016 and fitted them with ATS collars (G2110E (NeoLink), ATS, Isanti, MN, USA). We programmed collars to record locations every 30–60 min until September–November 2016. Prior to RSF model construction, we filtered the movement data to records collected between 1 May 2016 and 31 October 2016 (here, the estimated EHDV transmission period), then resampled them to a common sampling interval of 60 min. Notably, this period also excludes the hunting season on public lands for this region, reducing impacts of hunting pressure on the deer, which can affect resource selection [23,24]; hunting is only allowed in a single portion of the wild lands studied here and ranch hunting was not conducted during this tracking period. Capture information and tracking periods for each deer in our study are reported in Table S1. Deer capture and handling protocols were developed by JKB and ranch personnel and approved by the University of Florida’s Institutional Animal Care and Use Committee (UF IACUC Protocols #201508838 and #201609412 to JKB).

### 2.3. Explanatory Environmental Variables

We aimed to build a model that would apply to both populations, making comparison possible, so we built models for each deer population using environmental covariates applicable to both groups to quantify resource selection in each group. We selected seven environmental variables that described the conditions of the study ranch and the managed public lands of the wild population.

Land cover data were derived from version 3.2 of the Cooperative Land Cover map created and managed by the FWC and Florida Natural Areas Inventory (FNAI) [25]; details of the process and data are described in detail online (https://myfwc.com/research/gis/applications/articles/fl-land-cover-classification/). There were initially too many vegetation types for our research purposes, so we reclassified and regrouped several similar vegetation communities to create three binary vegetation raster surfaces [2], one for each land cover type we thought would be relevant to WTD in our study, and a fourth raster of other categories to be used as the reference type in the RSF. Broadly, we categorized three types of forest ranging from more open upland pine to dense canopy bottomland hardwood, each found across the study area and each known to support deer. Upland pine forests consist of widely spaced pine trees with a groundcover of shrubs, grasses, and herbs. Upland mixed hardwood–coniferous forests have open to partially closed canopies of various oak (*Quercus* sp.), hickory (*Carya* sp.), and pine (*Pinus* sp.) species and a dense ground layer of many species of grasses and forbs. Bottomland mixed forests occur in intermediate areas between swamps and uplands. These closed-canopy forests can be diverse with both deciduous and evergreen trees, including sweetgum (*Liquidambar styraciflua*), various pines (*Pinus* sp.) and oaks (*Quercus* sp.), and sweetbay (*Magnolia virginiana*) [26]. Land cover types in the rural/developed/other category included roads, water bodies, and manmade agricultural features such as pastures.

Three additional landscape variables were included in the RSFs: distance to tertiary roads, distance to permanent surface water bodies, and distance to supplemental food resources (feeders or food plots). We obtained state forest road data as shapefiles directly from the Florida Forest Service (FFS) and merged those with other road data from the study area derived by United States Geological Survey (USGS) and available through the Florida Geographic Data Library (FGDL; https://www.fgdl.org/metadataexplorer/explorer.jsp). As deer on the study ranch cannot access primary or secondary roads, we selected only tertiary roads (forest roads and small dirt roads with limited human access) from road data. Wild WTD in Florida have been found to avoid roads during the hunting season (Sep–Jan) more than non-hunting season [27], but we included the Euclidean distance of a point to the nearest tertiary road for comparison with ranched deer. We obtained data on permanent surface water sources from the National Hydrography Dataset available from the USDA Geospatial Data Gateway (https://datagateway.nrcs.usda.gov/). FWC provided us location data for supplemental food resources for wild deer, and the study ranch staff supplied similar data for ranched deer. We created distance-to-feature raster files in R [28] for these three environmental variables based on the tutorial provided by Meredith [29]. Briefly, we rasterized each dataset for each group of deer and calculated the distance of each cell containing no data to the nearest cell that did. We exported the resulting raster files as GeoTIFF files for analysis and projecting final models. For this study, all raster data were resampled to 10 m^2^ resolution. All distances were measured in meters.

After creating the distance-to-feature raster files for each group of deer in R, we followed the RSF procedure in Morris et al. [2] and Nekorchuk et al. [30]. For ranched deer, we first clipped the land cover raster files to the study ranch in ArcGIS Desktop 10.3.1 [31]. This represented the environmental resources free-ranging ranched deer could access. Second, we merged all the GPS points and randomly selected four points per deer per day to represent used points. Third, we created a 100% minimum convex polygon (MCP) around the used points to define the available area within which the deer could have selected resources [2,30]. Fourth, we randomly generated 5 times the number of used points within the available area to represent available points (i.e., pseudo-absences); all pseudoabsence points were drawn from the 100% MCPs. Finally, we coded used points as 1 and available as 0, then extracted the environmental values at each of these points. We repeated this five-step procedure for wild deer, except we did not limit the 100% MCP around the used points to the state-managed properties’ boundaries, as they are not a physical barrier equivalent to the high fences of the ranched property.

### 2.4. Resource Selection Model Development

We first screened variables prior to their inclusion in model generation for correlation with other variables in our study (Pearson’s correlation coefficient |*r*| ≥ 0.7 and significance in univariate general linear model analyses *p* < 0.1). Additionally, a variance inflation factor (VIF) analysis was conducted using the “usdm” package [32] to test for multicollinearity between variables. VIF values less than 10 were kept for model building. We generated a list of models including all additive combinations of covariates. Then, we standardized all continuous variables (distance to tertiary roads, permanent water bodies, and supplementary food resources) to allow for a direct comparison between model coefficients. Next, we fit generalized linear mixed models (GLMMs) to various combinations of covariates using the “lme4” R package [28,33]. A GLMM accounts for the unevenness in the number of animals in each study group and variability in the number of GPS data points collected from each deer by specifying a random intercept per individual. In our GLMMs, we included environmental variables as fixed effects and a random intercept for individual deer to determine whether variation between animals influenced the results of the models. After creating 56 models for each group of deer (112 total), we then generated spatial predictions of the probabilities of use by deer [19,20]. The RSFs were based on a logistic regression model in which a value of 1 represents a used resource and 0 available:

(1) $$w\left(x\right)=exp\left(\beta_{0}+\beta_{1}X_{1}+\ldots +\beta_{i}X_{i}\right)$$

where *w(x)* is the relative probability of a pixel being selected [19], *β_0_* is the intercept, and *β_i_* is the coefficient for variable *X_i_*. If *β_i_* is negative for the water, food resources, or tertiary roads variables, selection for that resource is indicated whereas a *β_i_* that is positive indicates avoidance of that resource [20]. The opposite is true for land cover classes. To account for variation among individual animals, we included a random intercept term in the mixed-effect models [34]. After completing model iteration for each group of deer, we used Akaike’s information criterion (AIC) to rank models by their ability to describe empirical data at the population level [35]. The AIC score identifies the most parsimonious model, which has the fewest variables explaining the greatest amount of variation [36]. We ranked models using the difference in AIC between the model of interest and the model with the smallest AIC (ΔAIC) [35,37] and calculated Akaike weights (*w_i_*) [35] to identify a set of competing models [38]. We defined competing models as all those required for *Σw_i_* to be >0.95, because the best model identified with an AIC approach is not necessarily the best representative of landscape use [38].

To select the final model, we compared the predictive accuracies of competing models [38] using a five-fold cross validation approach to determine the relationship between predicted selection and use [19]. For each fold model, we randomly selected and withheld 20% of the used GPS points from model creation. Then, we recreated the candidate models with unstandardized variables to calculate and map RSF values for each pixel, employing the Nelder–Meld optimizer algorithm for ranched deer [33,39] and the Nelder–Meld simplex optimizer algorithm from the “nloptr” R package [40] for wild deer to maximize and stabilize fold model fit. We selected these optimizers, as they had the smallest maximum gradient values (Tables S2 and S3). We split the resulting RSF values from each fold model into 10 equal area bins representing an equal proportion of the landscape and 10 equal bins of RSF values with the lowest bin rank (1) representing the lowest probability of selection and the highest bin rank (10) the greatest probability [41]. Next for each fold model, we calculated Spearman’s rank correlation coefficients (*ρ*) and variance in *ρ* between the number of points per bin and the bin rank before averaging these values to evaluate prediction success. A strong, positive *ρ* indicates a strong relationship between used locations and predicted selection [19]. We selected the final model for each group based on their respective fold models’ *ρ* and mapped the final model across the study area at a 10 m^2^ resolution by solving the logistic equation with unstandardized model coefficients, then splitting the RSF values into whichever method yielded the highest average *ρ*. We created our final maps of resource selection probability with ArcGIS Desktop 10.3.1 [34]. All final maps were at the 10 m^2^ resolution of the raster layers used in model development.

## 3. Results

### 3.1. Data Summary

We collected 72,841 GPS fixes from ranched and 22,159 from wild WTD for this study. After filtering movement data for points recorded during the EHDV transmission season and thinning them to an hourly sample interval, we had 46,818 fixes from ranched animals and 20,928 from wild deer.

### 3.2. Resource Selection Models

We evaluated correlation and collinearity between covariates ahead of performing RSF models. There were no two variables in the dataset with a Pearson’s correlation coefficient |r| ≥ 0.7, except for upland pine and bottomland mixed forest covers for ranched deer (*r* = −0.7068). Bottomland mixed hardwood forest was not significant for ranched WTD and distance to water was not significant for wild deer in univariate analyses (*p* < 0.1). We decided to keep these variables in model list development, because bottomland mixed hardwood forests comprise nearly one-third of the land cover on the study ranch, and many of the wild deer in our study were caught on state-managed lands near a major lake. Further, variance inflation factor (VIF) analysis values ranged from 1.075 to 2.873 for ranched animals and 1.068 to 2.388 for wild animals, indicating multicollinearity was not an issue with the included variables [42]. Thus, we created 56 RSF models for each group of deer from the additive combinations of all our covariates (excluding single variable models).

There were three competitive models to describe resource selection for ranched deer. The best model (ΔAIC = 0.00) for ranched deer included upland mixed hardwood–pine vegetation, bottomland mixed hardwood vegetation, distance from tertiary roads, distance from permanent water bodies, and distance from supplementary food resources. In contrast, the model including the full variable set was best (ΔAIC = 0.00) for predicting wild deer resource selection during the 2016 EHDV season (Table S4). Although other models for predicting resource selection by ranched deer had higher predictive accuracy (*ρ)* (Table 1), our goal was to compare resource selection between ranched and wild animals. Therefore, we chose to map the full variable set model for both ranched and wild deer. We list predictive accuracy of the best model for wild WTD resource selection in Table 2. We list random effects of ranched and wild individuals in Table S5.

In the final model for ranched animals, all the variables were significant at *α* = 0.05 except for the upland pine forest cover (*p* = 0.6316). Coefficient estimates for ranched WTD indicated that they selected areas with bottomland mixed forest cover (0.1925 ± 0.0442) and those closer to tertiary roads (−0.3331 ± 0.0151), permanent water bodies (−0.0825 ± 0.0145), and supplementary food resources (−0.0412 ± 0.0154) while avoiding upland pine forests (0.0202 ± 0.0421). All variables were significant at *α* = 0.05 in the final RSF model for wild WTD. Closer proximity to tertiary roads (−1.1258 ± 0.0389) and supplementary food resources (−0.1858 ± 0.0233) and greater distance away from permanent water bodies (0.6075 ± 0.0224) were associated with a higher predicted relative probability of selection by wild animals. Moreover, wild WTD selected upland pine forest (2.5568 ± 0.1208), upland mixed hardwood-coniferous forest (1.6507 ± 0.1234), and bottomland mixed hardwood forests (2.2778 ± 0.1220) over the rural/developed/other land cover class (−4.3384 ± 0.2899). Note, positive selection of covariates describing distance to features (e.g., distance to water) will have a negative coefficient. We list the coefficient estimates from the standardized all-inclusive models for both ranched and wild WTD in Table 2.

### 3.3. Spatial Predictions

**Figure 3:**
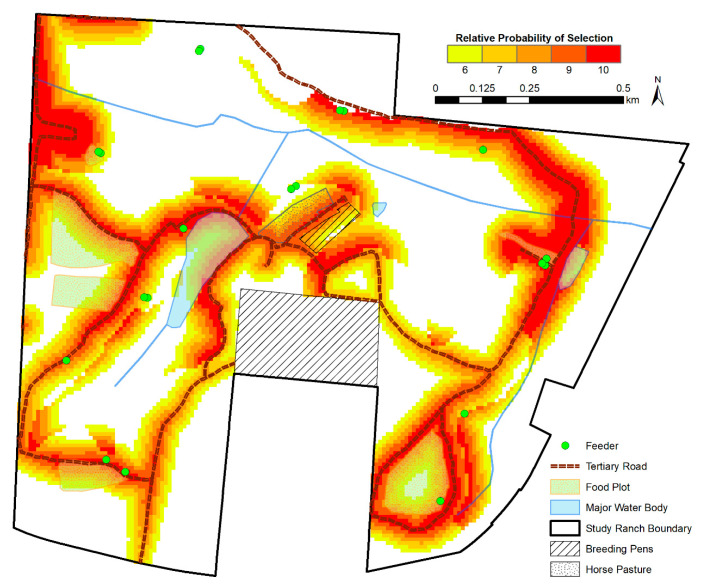
The relative probability of ranched white-tailed deer resource selection on the study ranch in panhandle Florida. The top 5 of 10 equal areas bins are depicted, with higher resource selection values representing higher predicted relative probability of selection.

**Figure 4:**
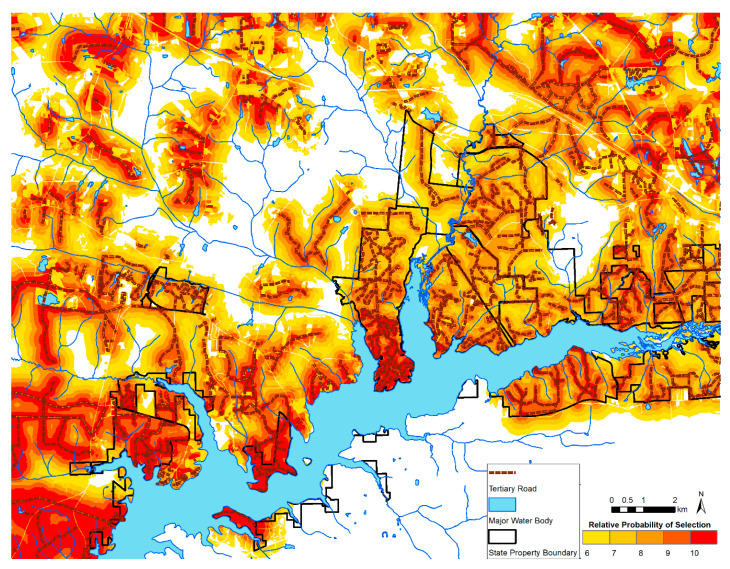
The relative probability of resource selection by wild white-tailed deer on state-managed properties near the study ranch in panhandle Florida. The top 5 of 10 equal areas bins are depicted, with higher resource selection values representing higher predicted relative probability of selection.

To map resources selected by WTD on the ranch and in the wild areas, we projected the top five bins of relative preference of selection onto the study area (6 or higher; Figure 3 and Figure 4, respectively). Generally, both maps show the highest probability of selection near tertiary roads. For wild WTD, there was a high likelihood of selection for areas bordering the major lake in the study area.

## 4. Discussion

EHD is an important disease of WTD in North America, yet its spatial patterns have not been well studied [4]. Here, we examined second-order resource selection differences in ranched and wild deer where we previously showed higher rates of EHDV exposure in those ranched deer [17]. For wild deer, the model including all the variables was best for estimating resource selection. Ranched deer had three competing models, including the all-inclusive model. As our main objective here was to compare differences in resource selection between ranched and wild deer during the 2016 EHDV season, we selected the all-inclusive model to predict resource use across the landscape for both wild and ranched deer. The predictive accuracy of the all-inclusive model for ranched WTD split by equal area bins was not the highest, so there is likely a better model for representing ranched deer resource selection. This suggests that wild and ranched deer experienced different life pressures that influence resource selection by these two groups.

Ranched and wild WTD appeared to use forested vegetation communities differently. Relative to the rural/developed/other land cover class, ranched deer selected bottomland mixed forests and avoided upland mixed hardwood–coniferous forests, although we did not find that they avoided upland pine forest. In contrast, we found that wild deer preferred all the land cover types nearly equally over rural/developed/other landscapes. WTD seek thermal cover in subtropical humid climates with hot summers [43], so both wild and ranched deer in our study may seek the cooler environment offered by closed vegetation types such as bottomland mixed forests during the day [44]. However, although the landscape compositions on the state-managed lands and private study ranch are roughly similar (upland pine and bottomland mixed hardwood forests ≈30% each, upland mixed hardwood–coniferous forest ≈15%, rural/developed/other ≈20%), high fences on the study ranch may be limiting the range ranched deer can select [45,46]. Thus, our study on state-managed properties may represent sampling at a larger scale than that on the study ranch.

Both ranched and wild WTD selected areas closer to supplementary food resources. However, the significance of this covariate was weaker for ranched compared to wild deer. Selection for these areas could be driven by forage quality, quantity, and efficiency [43,44]. WTD on state-managed properties were populated at a density of ≈0.08 animals/ha, whereas WTD on the study ranch were assessed at ≈1.48 animals/ha, not including all the non-native big game species co-habiting the ranch [17]. At high population density, intense intraspecific and interspecific competition for supplementary food resources on the study ranch may prevent ranched WTD from choosing areas closer to feeders [45,47]. Additionally, the staff at the study ranch noted that the food plots planted in 2016 were poor in quality and quickly became unpalatable to deer. In contrast, at low population density, intense competition is less likely, so wild deer can more freely select areas closer to supplementary food resources on state-managed lands. However, we do not know the nutritive quality of these food plots nor how attractive they were to wild WTD in 2016. Moreover, we captured several wild WTD near supplementary food resources (food plots), which may have inadvertently led us to study animals that prefer these areas.

Ranched deer selected areas closer to permanent water bodies, while wild WTD exhibited the opposite behavior. Ranched animals may be more reliant on permanent sources of standing water because lower availability of quality forage may prevent them from replenishing water from their food. A heavy dependence of supplementary protein feed may increase water requirements for ranched deer [48]. However, water requirements can be difficult to predict because of variability from weather conditions, energy expenditure, diet, growth and reproductive states, and many other factors [49]. Our model for resource selection by wild WTD indicates their avoidance of permanent water bodies, yet our final RSF map for this group predicted a high relative probability of selection for areas bordering the major lake in our study region. These seemingly contradictory results may be attributable to the nearness of many state-managed tertiary roads to the (large) lake. Moreover, this finding may be due in part to our capture of many wild on state-managed properties near the lake.

Finally, closer proximity to tertiary roads was significant to both ranched and wild WTD in our north Florida study area. This is reflected in our final maps. We may have discovered this amongst ranched deer, because most of the supplementary food resources are located along tertiary roads for ease of access by ranch staff. Furthermore, the tertiary roads cross through every major land cover type on the ranch, making the roads difficult for ranched deer to avoid. On state-managed lands, wild deer may be selecting spaces near tertiary roads because the roads allow access for forest management activities (timber harvest, tree thinning, etc.), which then produce a high amount of available forage that may benefit wild WTD [50].

Preference for bottomland mixed forests by ranched WTD coincides with the oviposition preferences of *Culicoides stellifer*, a suspected vector of EHDV in Florida [51]. Gravid biting midges of this species prefer to oviposit in substrates with mud and vegetation, such as edges of puddles and seepages readily found in bottomland mixed forests [52,53]. The greater EHDV burden amongst ranched WTD compared to their wild fellows might be explained by the former’s interactions with vectors in bottomland mixed forests. Additionally, preference for permanent water bodies by ranched WTD coincides with the preference of many biting midge vector species to dwell in and near wet soil by water bodies. Consequently, this may intensify vector-host interactions and contribute to greater viral exposure amongst ranched deer.

All the resource preferences we discovered amongst ranched deer may have operated synergistically to yield their greater EHD burden relative to their wild counterparts. More than half of the supplementary protein feeders on the study ranch were in bottomland mixed forest. The putative vectors of EHDV to WTD in the southeastern U.S., *Culicoides stellifer* and *Culicoides venustus* [51], have been reported to dwell in shaded areas with wet substrates and decaying vegetation (e.g., wet pasture areas, stream margins, cypress sloughs, etc.) [54]. Bottomland mixed forests near permanent water bodies such as those in our study areas may provide suitable habitat for these vectors. The tertiary roads on the private study ranch may funnel free-ranging ranched WTD towards feeders coincidentally located in bottomland mixed forests and near a permanent water body. When ranched deer utilize feeders in these areas, they may experience intensive interactions with EHDV-infectious *Culicoides* vectors. Wild deer may be at comparably lower risk of acquiring EHDV due to their avoidance of areas near permanent water bodies and greater freedom of choice in their range.

Vaccination of whole deer herds may be infeasible for mitigating EHD in Florida, so the most effective disease management strategies may need to focus on altering the environment at the landscape-scale, population-scale deer behavior, or both such that interactions between deer and infectious vectors are limited. One potential strategy for reducing EHD burden amongst both ranched and wild deer populations would be to change feeder placement and/or timing of feeding. Moving feeders away from bottomland mixed hardwood forests may reduce the probability that ranched deer select them, thus potentially lowering their encounter rate with vectors. Timing animal feeding to times of limited vector activity and low seasonal abundance patterns may also help reduce biting midge interactions with susceptible WTD. These strategies may also be pertinent to state WTD managers providing feeders for wild animals.

Our study was limited in sample size over only one season, so extrapolating our results to game farms in general and/or wild deer populations would be premature. Replication of this study is needed to confirm our findings. Moreover, additional research involving the control of factors such as sample population age/sex structure, immune status, animal stocking density, and presence of other livestock on a property would reveal to what extent differential EHDV exposure between ranched and wild WTD can be explained by resource selection behavior. Our study here represents a preliminary exploration of a poorly understood arboviral disease system, and much remains to be studied to be able to unquestionably show an increase in EHD amongst ranched deer.

## 5. Conclusions

In summary, EHD outbreaks can lead to severe economic and recreational losses in the southeastern U.S., but little is known of its ecology. We constructed RSF models to investigate if differences in resource selection behavior between ranched and wild WTD populations in panhandle Florida could help explain the higher disease burden amongst ranched animals. We showed that wild and ranched populations of WTD in panhandle Florida exhibit divergent movement behaviors, which could subsequently produce the high viral seroprevalence and antibody titer in ranched deer we previously recorded [17]. Additionally, we provide risk maps useful for targeting disease interventions in areas highly used by WTD hosts. This study reiterates that multiple environmental factors acting together can produce unique disease dynamics and prevalence in conspecific animal populations located near each other but separated by a physical barrier. Control strategies for vector-borne diseases in distinct animal populations should consider the special life pressures that influence each group’s habitat selection behavior.

## Figures and Tables

**Table 1:** Predictive accuracy (*ρ*) of competing models of ranched and wild white-tailed deer resource selection in the panhandle of Florida split by equal area bins. For each model, we report the mean Spearman rank correlation coefficient (*ρ_x̅_*), which measures the relationship between model prediction and use, and variance in *ρ* (*ρ_σ_*^2^) across 5 cross-validation folds. Bold text identifies the global model applied to both ranched and wild deer populations.

*Ranched*	Equal Area *ρ_x̅_*	Equal Area *ρ_σ_*^2^
Upland mixed hardwood–pine + bottomland mixed hardwood + tertiary roads + water + food	0.9654	0.0002
**Upland pine + upland mixed hardwood–pine + bottomland mixed hardwood + tertiary roads + water + food**	**0.9715**	**0.0002**
Upland mixed hardwood–pine + bottomland mixed hardwood + tertiary roads + water	0.9782	0.0004
*Wild*		
**Upland pine + upland mixed hardwood–pine + bottomland mixed hardwood + tertiary roads + water + food**	**0.9438**	**0.0002**

**Table 2:** Coefficient estimates ± standard error (SE) for the covariates included in the standardized final models predicting ranched and wild white-tailed deer resource selection in the panhandle of Florida study area during the 2016 epizootic hemorrhagic disease virus (EHDV) season.

Covariate	Ranched Estimate ± SE	Wild Estimate ± SE	Ranched *p*-Value	Wild *p*-Value
Intercept	−1.7281 ± 0.0612	−4.3384 ± 0.2899	<0.001	<0.001
Upland pine forest	0.0202 ± 0.0421	2.5568 ± 0.1208	0.6316	<0.001
Upland mixed hardwood–coniferous forest	−0.7400 ± 0.0835	1.6507 ± 0.1234	<0.001	<0.001
Bottomland mixed forest	0.1925 ± 0.0442	2.2778 ± 0.1220	<0.001	<0.001
Distance to tertiary roads	−0.3331 ± 0.0151	−1.1258 ± 0.0389	<0.001	<0.001
Distance to permanent water bodies	−0.0825 ± 0.0145	0.6075 ± 0.0224	<0.001	<0.001
Distance to supplementary food sources	−0.0412 ± 0.0154	−0.1858 ± 0.0233	0.0072	<0.001

## Data Availability

No new data were created or analyzed in this study. Data sharing is not applicable to this article.

## Supplementary Materials

The following are available online at https://www.mdpi.com/2076-2615/11/1/211/s1, Table S1: Deer collared for the 2016 comparative resource selection study, Table S2: Maximum gradient values of ranched deer unstandardized models with various optimizers, Table S3: Maximum gradient values of wild deer unstandardized models with various optimizers, Table S4: Competing models (Σ*w_i_* > 0.95) predicting ranched and wild deer resource selection during the 2016 EHDV transmission risk period (May–Oct) in the panhandle Florida study ranch, Table S5: Random effects from the final standardized RSF model for ranched and wild WTD studied during the 2016 EHDV season.
