# Supplementary material for: Resource Selection by Wild and Ranched White-Tailed Deer (Odocoileus virginianus) during the Epizootic Hemorrhagic Disease Virus (EHDV) Transmission Season in Florida
Source: Animals (Basel). 2021 Jan 16;11(1):211. doi: 10.3390/ani11010211 (PMC7830392; doi:10.3390/ani11010211)
Supplement: Supplementary file 1 [file animals-11-00211-s001.zip › Table S1.docx]

Table S1. Deer collared for the 2016 comparative resource selection study. †May 1 – Oct 31 = 184 days. ^Individuals collared beyond 2016-10-31.

| Deer ID | Sex | Wild or Ranched | Start Date | End Date | Collar Type | # days recorded during 2016 EHDV season^†^ | Random effects intercept from final RSF model |
| --- | --- | --- | --- | --- | --- | --- | --- |
| OV063 | F | Ranched | 2016-04-13 | 2016-10-03 | ATS Neolink | 156 | 0.1415 |
| OV061 | F | Ranched | 2016-04-13 | 2016-09-27 | ATS Neolink | 150 | 0.1030 |
| OV069 | F | Ranched | 2016-04-14 | 2016-10-04 | ATS Neolink | 157 | 0.1797 |
| OV070 | F | Ranched | 2016-04-14 | 2016-09-22 | Lotek 3300S | 145 | 0.0989 |
| OV068 | F | Ranched | 2016-04-14 | 2016-07-20 | Lotek 3300S | 81 | -0.4753 |
| OV064 | F | Ranched | 2016-04-14 | 2016-09-17 | ATS Neolink | 153 | 0.0315 |
| OV059 | F | Ranched | 2016-04-13 | 2016-09-21 | ATS Neolink | 144 | 0.0936 |
| OV102 | F | Wild | 2016-06-03 | 2016-06-29 | ATS Neolink | 27 | -1.2758 |
| OV165 | F | Wild | 2016-05-24 | 2016-11-10 | ATS Neolink | 161 | 0.2787 |
| OV154 | F | Wild | 2016-06-01 | ^ | ATS Neolink | 153 | 0.2171 |
| OV155 | F | Wild | 2016-06-01 | 2016-06-26 | ATS Neolink | 25 | -1.2201 |
| OV168 | F | Wild | 2016-06-22 | ^ | ATS Neolink | 132 | 0.3433 |
| OV067 | M | Ranched | 2016-04-14 | 2016-09-02 | Lotek 3300L | 103 | -0.2591 |
| OV074 | M | Ranched | 2016-04-15 | 2016-09-21 | Lotek 3300L | 144 | 0.0783 |
| OV073 | M | Ranched | 2016-04-15 | 2016-09-23 | Lotek 3300L | 146 | 0.0758 |
| OV066 | M | Ranched | 2016-04-14 | 2016-08-03 | Lotek 3300S | 95 | -0.2952 |
| OV065 | M | Ranched | 2016-04-14 | 2016-09-21 | Lotek 3300S | 144 | 0.0844 |
| OV062 | M | Ranched | 2016-04-13 | 2016-09-21 | Lotek 3300L | 144 | 0.0433 |
| OV071 | M | Ranched | 2016-04-14 | 2016-09-22 | Lotek 3300L | 145 | 0.0613 |
| OV072 | M | Ranched | 2016-04-14 | 2016-09-21 | Lotek 3300S | 144 | 0.0481 |
| OV169 | M | Wild | 2016-06-23 | ^ | ATS Neolink | 132 | 0.6719 |
| OV166 | M | Wild | 2016-06-21 | 2016-11-08 | ATS Neolink | 133 | 0.2365 |
| OV167 | M | Wild | 2016-06-20 | ^ | ATS Neolink | 134 | 0.7594 |
