# Supplementary material for: Resource Selection by Wild and Ranched White-Tailed Deer (Odocoileus virginianus) during the Epizootic Hemorrhagic Disease Virus (EHDV) Transmission Season in Florida
Source: Animals (Basel). 2021 Jan 16;11(1):211. doi: 10.3390/ani11010211 (PMC7830392; doi:10.3390/ani11010211)
Supplement: Supplementary file 1 [file animals-11-00211-s001.zip › Table S2.docx]

Table S2. Maximum gradient values of ranched deer unstandardized models with various optimizers.

| Optimizer | Maximum gradient value |
| --- | --- |
| NLOPT_LN_BOBYQA | 0.3477 |
| NLOPT_LN_NELDER_MEAD | 0.0317 |
| bobyqa | 0.3172 |
| Nelder_Mead | 0.0033 |
