# Supplementary material for: Resource Selection by Wild and Ranched White-Tailed Deer (Odocoileus virginianus) during the Epizootic Hemorrhagic Disease Virus (EHDV) Transmission Season in Florida
Source: Animals (Basel). 2021 Jan 16;11(1):211. doi: 10.3390/ani11010211 (PMC7830392; doi:10.3390/ani11010211)
Supplement: Supplementary file 1 [file animals-11-00211-s001.zip › Table S3.docx]

Table S3. Maximum gradient values of wild deer unstandardized models with various optimizers.

| Optimizer | Maximum gradient value |
| --- | --- |
| NLOPT_LN_BOBYQA | 0.4266 |
| NLOPT_LN_NELDER_MEAD | 0.1221 |
| bobyqa | 0.2904 |
| Nelder_Mead | 0.1222 |
