# Supplementary material for: Resource Selection by Wild and Ranched White-Tailed Deer (Odocoileus virginianus) during the Epizootic Hemorrhagic Disease Virus (EHDV) Transmission Season in Florida
Source: Animals (Basel). 2021 Jan 16;11(1):211. doi: 10.3390/ani11010211 (PMC7830392; doi:10.3390/ani11010211)
Supplement: Supplementary file 1 [file animals-11-00211-s001.zip › Table S4.docx]

Table S4. Competing models (Σ*w_i_* > 0.95) predicting ranched and wild deer resource selection during the 2016 EHDV transmission risk period (May-Oct) in the panhandle Florida study ranch. These models are derived from the model list of all additive combinations of variables. For each model, we report model covariates, change in Akaike’s Information Criterion (ΔAIC), and AIC weights (*w_i_*).

| Ranched | ΔAIC | *w_i_* |
| --- | --- | --- |
| Upland mixed hardwood-pine + bottomland mixed hardwood + tertiary roads + water + food | 0.00 | 0.66 |
| Upland pine + upland mixed hardwood-pine + bottomland mixed hardwood + tertiary roads + water + food | 1.77 | 0.27 |
| Upland mixed hardwood-pine + bottomland mixed hardwood + tertiary roads + water | 5.00 | 0.05 |
| Wild |  |  |
| Upland pine + upland mixed hardwood-pine + mixed bottomland hardwood + tertiary roads + water + food | 0.00 | 1.00 |
