# Supplementary material for: Resource Selection by Wild and Ranched White-Tailed Deer (Odocoileus virginianus) during the Epizootic Hemorrhagic Disease Virus (EHDV) Transmission Season in Florida
Source: Animals (Basel). 2021 Jan 16;11(1):211. doi: 10.3390/ani11010211 (PMC7830392; doi:10.3390/ani11010211)
Supplement: Supplementary file 1 [file animals-11-00211-s001.zip › Table S5.docx]

Table S5. Random effects from the final standardized RSF model for ranched and wild WTD studied during the 2016 EHDV season.

| Ranched Deer ID | Intercept | |
| --- | --- | --- |
| OV063 | 0.1415 | |
| OV061 | 0.1030 | |
| OV067 | -0.2591 | |
| OV069 | 0.1797 | |
| OV070 | 0.0989 | |
| OV074 | 0.0783 | |
| OV073 | 0.0758 | |
| OV066 | -0.2952 | |
| OV065 | 0.0844 | |
| OV068 | -0.4753 | |
| OV062 | 0.0433 | |
| OV071 | 0.0613 | |
| OV072 | 0.0481 | |
| OV064 | 0.0315 | |
| OV059 | 0.0936 | |
| Wild Deer ID |  |  |
| OV102 | -1.2758 |  |
| OV165 | 0.2787 |  |
| OV154 | 0.2171 |  |
| OV155 | -1.2201 |  |
| OV169 | 0.6719 |  |
| OV166 | 0.2365 |  |
| OV168 | 0.3433 |  |
| OV167 | 0.7594 |  |
